# 31.1 OPTIMISING THE TREATMENT AND MANAGEMENT OF FIRST-EPISODE SCHIZOPHRENIA: THE OPTIMISE CLINICAL TRIAL

**DOI:** 10.1093/schbul/sby014.127

**Published:** 2018-04-01

**Authors:** René Kahn, OPTiMISE Study Group

**Affiliations:** Icahn School of Medicine at Mount Sinai

## Abstract

**Background:**

Very few prospective, sequential studies are available that could guide decisions which have to be made in every day clinical routine. Some of the simplest questions of clinicians remain unanswered. For example: If the first antipsychotic used has not worked, is switching to another drug effective? Or should we perhaps increase the dose? And when should we start clozapine, the most efficacious drug? These questions are most urgent for patients with a first episode of psychosis.

**Methods:**

OPTiMiSE is an international clinical trial, conducted in 27 research centers located in 14 countries across Europe and Israel. Among other purposes, OPTiMiSE was designed to test a three-stage treatment algorithm. The 446 participants of OPTiMiSE were diagnosed with a first episode of schizophrenia, schizophreniform or schizoaffective disorder. Each participant started with a 4-week open label amisulpride treatment. After these 4 weeks, non-remitters are randomized to switch to another antipsychotic versus continuation of amisulpride for 6 weeks, in a double-blind fashion (phase 2). After these additional 6 weeks, non-remitters were switched to clozapine for another 12 weeks.

**Results:**

Description of the sample:

Mean age at inclusion was 26 years, 30% of the patient sample was female. The median duration of illness was 4 months and none of the participants had been using antipsychotic medication for more than 2 weeks. All patients were treated on a voluntarily basis. The patient sample was moderately ill at baseline, with a mean score on the Positive And Negative Syndrome Scale (PANSS) of 78 (sd 19).

Phase 1

Patients started on 200–800 mg/day amisulpride treatment, with a target dose of 400 mg/day. Drop-out rate was 20%. Out of the 446 patients who were initiated on amisulpride, 56% met remission criteria, based on the eight remission items of the PANSS. Side effects were mild, with a mean weight increase of 2.7 kg (sd 3.3).

Phase 2

At the end of phase 1, 121 patients did not meet remission criteria. Out of the 93 patients who continued into the double-blind treatment phase, 72 patients completed the 6-week treatment phase. Remission rate in phase 2 was 35%. There was no significant difference between the two treatment arms regarding remission rate, nor regarding the drop out rate. Patients randomized to olanzapine gained significantly more weight compared to amisulpride.

Phase 3

At the end of phase 2, 40 patients did not meet remission criteria. Out of the 28 patients who continued into the open-label clozapine treatment phase, 18 patients completed this 12-week treatment from which only 5 patients met remission criteria, translating into a remission rate of only 18%. Non-remitters generally did improve to a great extent but failed to meet the stringent remission criteria.

**Discussion:**

Amisulpride is confirmed to be a good option for initiating pharmacotherapy in first episode patients, resulting in a high remission rate. There was no advantage related to remission rates or drop out rates for switching non-responders after 4 week of treatment to another antipsychotic versus continuing the initiated antipsychotic for another 6 weeks; continuing the first antipsychotic may have the benefit of avoiding new side effects related to introducing a new antipsychotic. Despite a low remission rate following clozapine treatment, non-remitters generally did show a substantial reduction in symptoms, demonstrating that clozapine should be an option for non-responding psychotic patients early in the illness.

